# Recyclable High-Performance Epoxy-Anhydride Resins with DMP-30 as the Catalyst of Transesterification Reactions

**DOI:** 10.3390/polym13020296

**Published:** 2021-01-18

**Authors:** Wenzhe Zhao, Le An, Shujuan Wang

**Affiliations:** 1 State Key Laboratory for Strength and Vibration of Mechanical Structures, School of Aerospace Engineering, Xi’an Jiaotong University, Xi’an 710049, China; wenzhe0301@stu.xjtu.edu.cn; 2 School of Chemistry, Xi’an Jiaotong University, Xi’an 710049, China; shujuanwang@mail.xjtu.edu.cn

**Keywords:** epoxy resins, recycling, tertiary amines, transesterification reactions

## Abstract

Epoxy-anhydride resins are widely used in engineering fields due to their excellent performance. However, the insolubility and infusibility make the recycling of epoxy resins challenging. The development of degradable epoxy resins with stable covalent networks provides an efficient solution to the recycling of thermosets. In this paper, 2,4,6-tris(dimethylaminomethyl)phenol (DMP-30) is incorporated into the epoxy-glutaric anhydride (GA) system to prepare high-performance epoxy resins that can be recycled below 200 °C at ordinary pressure via ethylene glycol (EG) participated transesterification. The tertiary amine groups in DMP-30 can catalyze the curing reaction of epoxy and anhydride, as well as the transesterification between ester bonds and alcoholic hydroxyl groups. Compared with early recyclable anhydride-cured epoxy resins, the preparation and recycling of diglycidyl ether of bisphenol A (DGEBA)/GA/DMP-30 systems do not need any special catalysts such as TBD, Zn(Ac)_2_, etc., which are usually expensive, toxic, and have poor compatibility with other compounds. The resulting resins have glass transition temperatures and strengths similar to those of conventional epoxy resins. The influences of GA content, DMP-30 content, and temperature on the dissolution rate were studied. The decomposed epoxy oligomer (DEO) is further used as a reaction ingredient to prepare new resins. It is found that the DEO can improve the toughness of epoxy resins significantly. This work provides a simple method to prepare readily recyclable epoxy resins, which is of low-cost and easy to implement.

## 1. Introduction

Epoxy thermosets are widely used in electronic packaging, coatings, adhesives, composites, and other fields due to their excellent electrical insulation, high-temperature resistance, mechanical properties, and chemical stability [1,2,3,4,5]. However, once cured and shaped, epoxy thermosets cannot be melted or dissolved in organic solvents, which makes them difficult to recycle. The widespread application of epoxy thermosets has brought a serious concern about their large amounts of waste, including leftover pieces, off-grade products, and end-of-service-life components. The initial disposal methods, including landfill and incineration, led to severe environmental pollution and waste of resources [6,7,8,9]. To achieve the recycling of epoxy resins, various technologies have been developed, such as pyrolysis, supercritical fluid method, and chemical solvolysis [7,10,11]. However, these methods usually demand harsh conditions such as high temperature (250–450 °C), high pressure (3–27 MPa), and trenchant chemicals (NaOH, HNO_3_, etc.) [11,12,13,14]. These issues usually consume a lot of energy and require expensive custom equipment, which limits the wide application of epoxy thermosets recycling in the industry.

The development of vitrimers containing dynamic covalent bonds (DCBs) makes it possible to recycle thermosets under mild conditions [8,9,15,16,17,18,19,20,21,22,23,24,25]. Over the past few decades, researchers have developed an array of vitrimers through incorporating various DCBs (e.g., disulfide [25,26], imine-amine exchange [27], and hindered urea bond [28]), among which epoxy vitrimer prepared from the curing reactions of epoxy-anhydride [29,30,31] or epoxy-carboxylic [32,33,34] is the most investigated vitrimer system. The network topology can be rearranged via transesterification reactions (TERs) between ester bonds and hydroxyl groups at elevated temperatures, leading to the unique properties of materials such as malleability, self-healing, or reprocessability [21,29,35,36,37,38]. Furthermore, the TERs activated by proper catalysts and solvents were used as mechanisms for the dissolution and recycling of epoxy resins [22,34,39]. For example, Yu and Shi et al. [32,34] achieved nearly 100% recycling of the epoxy-fatty-acid-Zn(Ac)_2_ system using ethylene glycol (EG), and the repolymerized resin exhibited almost the same properties as that of the original resin. Further, industrial epoxy thermosets and composites can be dissolved in TBD-alcohol solvents via catalyst-activated TERs [40,41].

In order to activate TERs between ester bonds and alcoholic hydroxyl groups, sufficient high-efficiency catalysts such as Zn(Ac)_2_ [20,42], Sn(Oct)_2_ [43], TBD [44], and DBU [45] are necessary. However, the use of these special catalysts causes a series of problems, which greatly limits the wide application of epoxy vitrimers. Firstly, the commonly used catalysts are usually not bound in the network and have poor compatibility with organic compounds [40]. High catalyst loading may lead to the aggregation and leaching of catalysts, resulting in a decrease in the material performance [16,46]. Secondly, these catalysts are toxic and corrosive, which may cause corrosion damage to materials [47]. Thirdly, these catalysts are prone to oxidation failure at elevated temperatures and difficult to separate from the dissolution mixture, which limits the recycling of catalysts for reuse [41,48]. Moreover, most of the above-mentioned catalysts are expensive, leading to high preparation and recovery costs.

Recently, researchers developed a new type of catalyst-free epoxy vitrimers by introducing abundant hydroxyl groups or covalently bonded tertiary amines as the internal catalyst of TERs [46,47,49,50]. However, these polymers usually had the problems of complex synthesis processes, low glass transition temperatures (*T*_g_), and low strengths. Hao et al. [51] incorporated triethanolamine (TEOA) as a catalytic co-curing agent into the traditional epoxy-anhydride system to prepare recyclable epoxy vitrimer with high *T*_g_ and strength. The resulting resins can be easily hydrolyzed using a phosphotungstic acid aqueous solution. Here, the tertiary amines in TEOA played a dual role, which catalyzed both the curing reaction and the TERs between ester bonds and hydroxyl groups. This work also provides a new strategy to develop recyclable high-performance epoxy resins with low cost from industrial raw materials.

**Figure 1:**
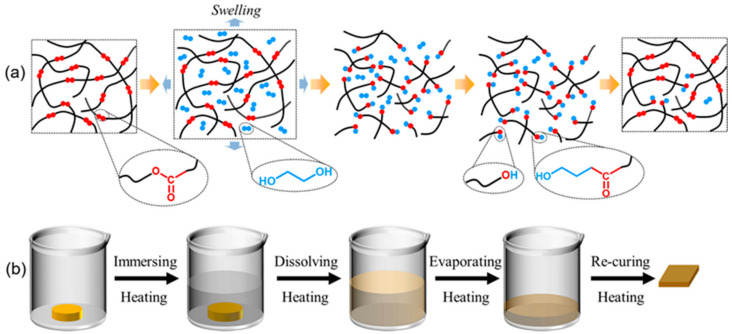
(**a**) The schematic of ethylene glycol (EG)-assisted dissolution and recycling mechanism: The crosslinked network is immersed in EG and heated, then alcohol molecules diffuse into the network and cleave ester bonds on the polymer skeleton. The repolymerization of decomposed epoxy oligomer occurs via evaporating excess EG. (**b**) The schematic illustration of the dissolution and recycling experiments.

One of the typical accelerators for epoxy resins is 2,4,6-tris(dimethylaminomethyl)phenol (DMP-30), which can effectively reduce the curing temperature and speed up the curing process [8,52]. It can also be used alone as the curing agent [53]. However, there have been no reports on the recycling of epoxy resins with DMP−30 as the catalyst of TERs. In this work, DMP-30 was introduced into epoxy-glutaric anhydride (GA) to prepare industrial-grade epoxy resins, which can be dissolved in ethylene glycol (EG) without any catalyst below 200 °C at ordinary pressure. Here, DMP-30 plays a dual role, whose tertiary amines and hydroxyl groups are involved in the curing reaction of epoxy-GA, and the tertiary amines can catalyze TERs between ester bonds and alcoholic hydroxyl groups. Compared with catalysts (TBD, Zn(Ac)_2_) used in early recyclable epoxy vitrimers reported in the literature, the introduction of DMP-30 avoids the disadvantages of high cost, toxicity, and poor miscibility with the epoxy-anhydride system. In this paper, EG is selected as the solvent for dissolution because of its high boiling point and rich hydroxyl groups (Table S1). The dissolution and recycling mechanism is based on the TERs between ester bonds in the network and hydroxyl groups in EG, which is illustrated in Figure 1a: when an epoxy sample is immersed in EG and heated, the EG molecules diffuse into the network, and the network swells; at the same time, alcoholic hydroxyl groups attack the ester bonds on the polymer skeleton, which breaks the long polymer chains into short segments, resulting in the degradation of the network; after the dissolution, the excessive EG is evaporated, and the dissolved resins can be reconnected to form new crosslinked networks, in which there will be some residual alcohol molecules and broken covalent bonds. The TERs in the process are catalyzed by tertiary amines. Figure 1b showed the schematic view of the recycling process. The effect of GA content, DMP-30 content, and temperature on the dissolution rate was studied. The reclaimed dissolved oligomer was used as a reaction ingredient to prepare new networks.

## 2. Materials and Methods

### 2.1. Materials

The epoxy oligomer diglycidyl ether of bisphenol A (DGEBA, M_W_: 340.41 g/mol) was purchased from Sigma Aldrich (St. Louis, MO, USA). The curing agent glutaric anhydride (GA, M_W_: 114.10 g/mol) and catalyst 2,4,6-tris(dimethylaminomethyl)phenol (DMP-30, M_W_: 265.39 g/mol) were ordered from Macklin Biochemical Co., Ltd. (Shanghai, China). The release agent JD-909 was obtained from Jiadan Mold Release Agent Co., Ltd. (Dongguan, China). Organic solvents used for swelling and dissolution tests including *N*,*N*-dimethylformamide (DMF, AR grade), 1,2,4-trichlorobenzene (TCB, AR grade), xylenes (AR grade), tetrahydrofuran (THF, AR grade), heptane (AR grade), and ethylene glycol (EG, AR grade) were provided by Macklin Biochemical Co., Ltd. (Shanghai, China). All the chemical reagents were used directly without any purification.

### 2.2. Preparation of Epoxy Resins

Epoxy groups were provided from DGEBA, and acyl groups were provided from GA. Five kinds of epoxy resins with different contents of curing agent were prepared. The formulations of epoxy resins were listed in Table 1. The stoichiometric ratio of an epoxy group and acyl group was defined as *r*, which varied between 1:1 and 1:2, while the DMP-30 content was kept at 2 wt% of the total weight of the curing system.

The epoxy resin samples were synthesized according to the following procedure. Firstly, DGEBA was heated at 130 °C to reduce the viscosity, while GA in solid-state was heated at 130 °C to melt completely. Secondly, the liquid GA was poured into DGEBA in proportion quickly and stirred at 130 °C for about 10 min to make the mixture homogeneous. Thirdly, DMP-30 was added to the beaker. The mixture was then stirred magnetically at 80 °C for about 3 min. Finally, the mixture was put into a vacuum to remove the bubbles and then poured into the home-made metal molds sprayed with a release agent. The reactive mixture was cured with a two-step program: 100 °C for 2 h, and 150 °C for 6 h. After curing, the samples were cooled naturally to room temperature. To prepare dumbbell-like, bar, and cylindrical samples, the curing was performed in metal molds with the corresponding shape.

### 2.3. Characterizations

#### 2.3.1. Differential Scanning Calorimeter (DSC)

DSC experiments were carried out with a DSC1 tester (Mettler Toledo, Zurich, Switzerland) to determine the curing conditions of the DGEBA/GA/DMP-30 curing system. In each case, a sample weighing ~10 mg was sealed in a 40 µL aluminum crucible. The sample was first stabilized at 0 °C for 10 min and then heated from 0 °C to 250 °C at the heating rate of 10 °C/min under a nitrogen atmosphere.

#### 2.3.2. Fourier Transform Infrared Spectroscopy (FTIR)

In order to monitor the functional groups of samples during the curing and recycling process, FTIR spectra were recorded by a Nicolet iS50 FTIR spectrometer (Thermo Fisher Scientific, Waltham, MA, USA) in attenuated total refraction (ATR) mode. Before testing, the sample was irradiated under an infrared lamp to remove the moisture. The dried sample was scanned from 650 to 4000 cm^−1^ for 32 scans with a resolution of 4 cm^−1^. The band of carbon-carbon stretching vibration of phenyl (*ν_c-c_*_(*ph*)_ = 1607 cm^−1^) was used as a reference to normalize the obtained spectra.

#### 2.3.3. Dynamic Mechanical Analysis (DMA)

DMA experiments were conducted on a DMA242E instrument (Netzsch, Selb, Germany) to obtain the thermal-mechanical behaviors of epoxy resins. The samples were cut into sheets with a dimension of 20 mm × 5 mm × 1 mm. During the experiments, the tensile mode was used at the frequency of 1 Hz, and the temperature increased isothermally from ambient temperature to 150 °C at the rate of 3 °C/min. Storage modulus (*E*’) and damping factor (tan δ) were collected during the temperature scan. The *T*_g_ value was assigned at the maximum of tan δ.

The crosslink density (*ν*) was calculated by Equation (1):

*ν* = *E*’/3*RT*, (1)

where *E*’ is the storage modulus of the resin in the rubbery plateau region at *T*_g_ + 50 °C, *R* is the gas constant, and *T* refers to the absolute temperature in Kelvin.

#### 2.3.4. Uniaxial Tension Tests

The mechanical properties of epoxy resins were characterized by uniaxial tension tests at room temperature, which were performed using an electronic universal testing machine (MTS Criterion C45.105, Eden Prairie, MN, USA). The samples were cured in dumbbell-like molds designed according to ASTM D638, and the effective size of the sample is 50 mm × 13 mm × 3~5 mm. The tensile rate was 1 mm/min for all the tests, and at least three specimens were tested in each sample.

#### 2.3.5. Thermogravimetric Analysis (TGA)

TGA measurements were performed on a simultaneous thermal analyzer (Netzsch STA449F5, Selb, Germany) in the TGA mode to evaluate the thermal stability of epoxy resins. The sample weighing ~10 mg was placed into an alumina pan and heated from ambient temperature to 800 °C at a heating rate of 10 °C/min under a nitrogen atmosphere with a gas flow rate of 50 mL/min.

### 2.4. Swelling and Dissolution Tests

The solubility of epoxy resins in DMF, TCB, xylenes, THF, and heptane at ambient temperature or high temperature was investigated. The sheet sample (*m*_0_) of 5 mm in length, 3 mm in width, and 2 mm in thickness was immersed in the organic solvent at room temperature for 96 h or 180 °C for 12 h, then taken out and weighed (*m*_s_). The swollen sample was then placed in a vacuum oven at 180 °C for 12 h to eliminate the solvent. The residual mass (*m*_d_) of the dried sample was recorded.

For the dissolution of epoxy resins in EG, the cylindrical specimen with a diameter of 10 mm and a height of 3 mm was soaked in EG entirely and sealed in a glass bottle with an aluminum cap. The mass ratio of the epoxy sample to EG is 1:25. The glass bottle was then placed in an oven, which had been heated to the designed temperature (140–200 °C). The selected temperature was around at the boiling point of EG and far less than the pyrolysis temperature of epoxy resins. At different time intervals, the sample was taken out, cleaned with 95% ethanol, and weighed immediately to record the mass change. The disappearance of solid epoxy resins meant the complete dissolution of samples. A mixture solution composed of epoxy oligomer and extra EG was derived.

### 2.5. Reuse of Decomposed Epoxy Oligomer

The recycling of decomposed epoxy oligomer (DEO) was achieved by heating the dissolved solution at a higher temperature (200 °C) in an open environment for ~12 h, where residual EG is evaporated. There were two disposal methods for recycled DEO. On the one hand, the DEO was cured directly at 180 °C for ~24 h to obtain the re-crosslinked network. Another strategy was to mix the DEO with the raw epoxy materials to prepare new resins. The specific synthesis process was as follows. The DEO (10 wt%, 20 wt% on the weight of epoxy resin curing system, respectively) and molten DGEBA were mixed at 130 °C for 10 min under magnetic stirring. After getting a homogeneous mixture, GA and DMP-30 were added successively in a determined amount and continuously stirred at 80 °C to obtain a uniform dispersion. After vacuuming, the reactive mixture was poured into the metal mold and cured with the same conditions as the original epoxy resins.

## 3. Results and Discussion

### 3.1. Preparation and Structural Characterization of Fresh Epoxy Resins

**Figure 2:**
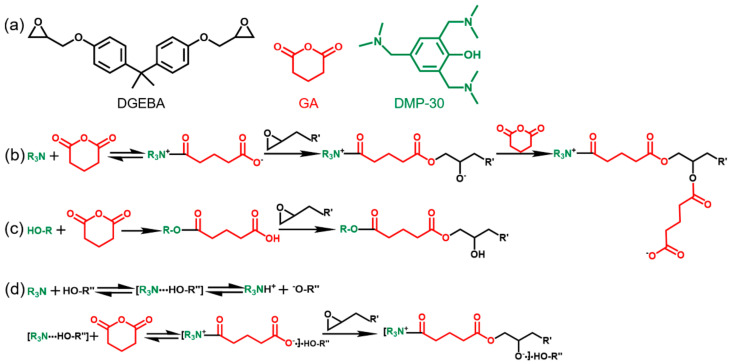
Curing reaction mechanisms of DGEBA and GA under the catalysis of DMP-30: (**a**) Detailed chemical structures of DGEBA, GA, and DMP-30; (**b**) The tertiary amine is able to open the anhydride ring to form carboxylate anion, which then opens the epoxy ring to yield ester and new alkoxide anion. The new anion then reacts with another anhydride; (**c**) The hydroxyl opens the anhydride ring to form a carboxylic group, which further reacts with the epoxy group and generate a new hydroxyl; (**d**) The tertiary amine, nonphenolic hydroxyl, and anhydride form a complex, which enhances the reactivity between the epoxy group and the anhydride.

The catalyzed curing of epoxy-anhydride systems is usually complicated [52,54]. Here we consider that the copolymerization between epoxy and anhydride in the presence of DMP-30 is the main reaction during the curing process (Figure 2) [55,56]. Detailed chemical structures of DGEBA, GA, and DMP-30 were plotted in Figure 2a. The role of tertiary amine groups in DMP-30 is to participate in the formation of carboxylate and alkoxide anions, thereby initiating the alternating copolymerization between anhydride and epoxy groups (Figure 2b) [53]. The anhydride rings were opened by the hydroxyl in DMP-30 to form the carboxylic acid groups, which can further react with the epoxy groups and generate a new hydroxyl (Figure 2c) [51]. When the tertiary amine and nonphenolic hydroxyl coexisted in the curing system, they formed a complex with the anhydride, which further enhanced the reactivity between the epoxy group and the anhydride (Figure 2d) [57]. When anhydride is insufficient in the system, homopolymerization and/or etherification of epoxy monomer may occur at high temperature [24], which is unfavorable to the dissolution of epoxy resins. Here we think that the copolymerization of epoxy and anhydride forming ester bonds is dominant, and the effects of secondary reactions are not considered. In summary, with the participation of DMP-30, a crosslinked network structure containing a large number of ester bonds was formed by the curing reaction of anhydride and epoxy groups.

**Figure 3:**
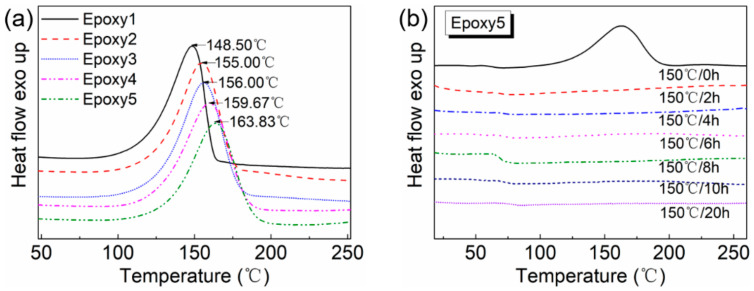
(**a**) Differential Scanning Calorimeter (DSC) curves of five uncured epoxy resin systems with different GA contents at the heating rate of 10 °C/min under a nitrogen atmosphere; (**b**) DSC curves of Epoxy5 after pre-cured at 100 °C for 2 h and post-cured at 150 °C for different hours at the heating rate of 10 °C /min under a nitrogen atmosphere.

DSC and FTIR tests were conducted to trace the curing process of epoxy resins and determine the curing condition. The DSC curves of uncured epoxy systems revealed that the curing reaction of DGEBA/GA/DMP-30 began at approximately 80–100 °C. With the increase of GA loading, the exothermic peak temperature shifted from 148.50 °C to 163.83 °C, while the end temperature was postponed gradually (Figure 3a). Therefore, we chose 100 °C as the pre-curing temperature and 150 °C as the post-curing temperature. To determine the curing time of the DGEBA/GA/DMP-30 systems, DSC tests were then performed using Epoxy5 as samples, which were pre-cured at 100 °C for 2 h and post-cured at 150 °C for different hours. The results showed that the exothermic peak of Epoxy5 disappeared after curing at 150 °C for 2 h (Figure 3b), indicating the completion of the curing reaction. The DSC results were consistent with the FTIR spectra, in which the ester bond peak (*ν_c=o_*_(*ester*)_ = 1732 cm^−1^) did not increase after curing at 150 °C for 2 h (Figure S1). Nonetheless, in the follow-up study, all samples were pre-cured at 100 °C for 2 h and then post-cured at 150 °C at least for 6 h to ensure sufficient ester bonds in the crosslinked networks.

**Figure 4:**
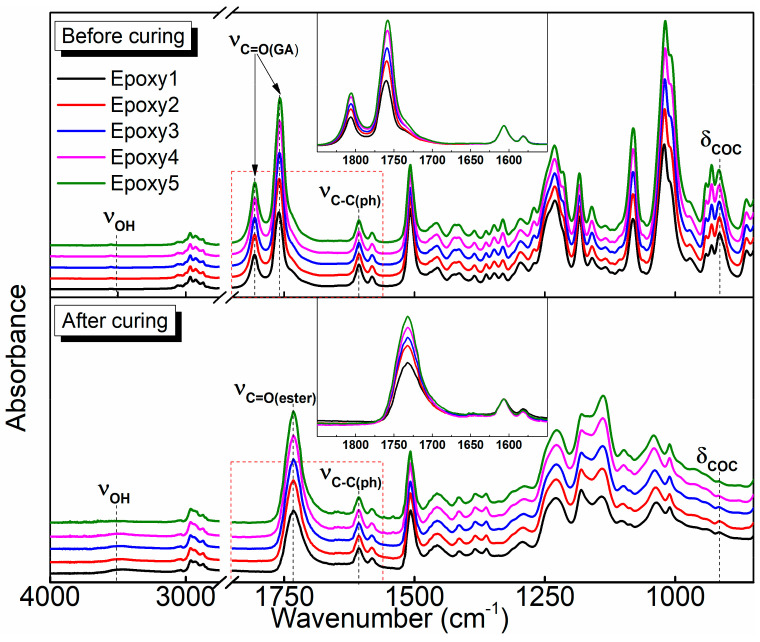
Fourier Transform Infrared Spectroscopy (FTIR) spectra of five epoxy systems with different contents of GA before and after curing. The curing condition is 100 °C for 2 h and then 150 °C for 6 h.

The chemical structures of five kinds of epoxy resins before and after curing were characterized by FTIR spectra (Figure 4). After curing, the stretching vibration peaks at 1806 cm^−1^ and 1759 cm^−1^ were attributed to carbonyl groups in GA disappeared, indicating that the anhydride groups completely reacted. However, the conversion of epoxy groups was not completed, and the residual unreacted moieties decreased with increasing the GA content, which was indicated by the intensity change of the characteristic absorption peak of the epoxide group (*δ_coc_*_(*epoxy*)_ = 914 cm^−1^). With the increase of GA content, the intensity of the ester bond peak increased, suggesting a higher content of ester bond in the crosslinked network. As the curing reaction tended to be complete with increasing the content of the curing agent [29,58], a visible hydroxyl peak at 3500 cm^−1^ appeared in the FTIR spectra when the ratio of epoxide to acyl, r = 1:1, while it disappeared when r = 1:2. Abundant hydroxyl groups are necessary for the TERs [29,51], but the number of hydroxyl groups in DGEBA/GA/DMP-30 is small. Therefore, the polymers are expected to be more stable, and correspondingly, the stress relaxation time is longer (Figure S2). However, this may not affect the TERs during the dissolution of polymers because the alcohol solvents can provide sufficient extra hydroxyl groups [59]. The results indicated that the DGEBA/GA/DMP-30 networks were rich in ester bonds, and the content of functional groups was closely related to the amount of curing agent, which will have a great effect on the properties of epoxy resins and their solubility in organic solvents [60,61,62,63,64].

### 3.2. Thermal and Mechanical Properties of Epoxy Resins

**Figure 5:**
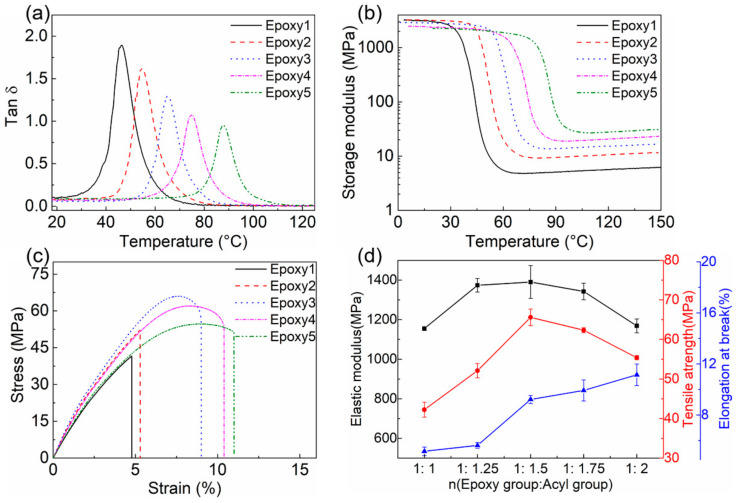
(**a**) Tan δ curves as a function of temperature. Take the temperature corresponding to the peak of the curve to be *T*_g_; (**b**) Storage modulus curves of epoxy resins with different GA contents as a function of temperature; (**c**) Uniaxial tensile stress-strain curves of fresh epoxy resins; (**d**) The changes of elastic modulus, tensile strength, and elongation at break with the ratio of the epoxy group and acyl group.

The dynamic mechanical properties of epoxy resins were characterized by DMA tests. *T*_g_s obtained from the peak temperature of tan δ curves were changed from 46.39 °C to 87.73 °C (Figure 5a). The storage modulus as a function of temperature for each sample indicated the elastic responses of materials (Figure 5b). The rubbery modulus (storage modulus in the plateau section) was used to calculate the crosslink density according to Equation (1). As the amount of GA increased, the rubbery modulus shifted from 5.28 MPa to 29.86 MPa, and the crosslink density ranged from 0.57 mmol/cm^3^ to 2.91 mmol/cm^3^. These results were summarized in Table 2. The changes in temperature-related mechanical properties can be attributed to the differences in the chemical structure of the five samples. With the increase of crosslink density, the movement of polymer chains became more difficult, resulting in the elevated rubbery modulus and *T*_g_s [61,65].

Figure 5c plotted the stress-strain curves of five epoxy resins, and the changes in elastic modulus, tensile strength, and elongation at break were summarized in Figure 5d. With the increase of GA content, the elastic modulus and tensile strength first increased and then decreased slightly. Epoxy3 with *r* = 1:1.5 exhibited the highest tensile strength (65.59 MPa) and elastic modulus (1390.24 MPa). Epoxy1 with the least amount of GA showed the lowest tensile strength (42.24 MPa) and elastic modulus (1155.05 MPa). The elongation at break increased gradually with *r*. The difference in the mechanical properties of epoxy resins can be attributed to the difference in crosslink density and the number of unreacted groups in networks [63,66,67,68,69]. The free moieties, including anhydride groups, epoxy groups, and tertiary amines, played a role in plasticizing, reducing the tensile strength and elastic modulus [69]. Compared with the other degradable epoxy resins based on TERs (Table S2) [39,50,59,70], the epoxy resins prepared by DGEBA/GA/DMP-30 exhibited better mechanical properties. We can flexibly adjust the properties of the resins by selecting the GA content to satisfy different application requirements.

**Figure 6:**
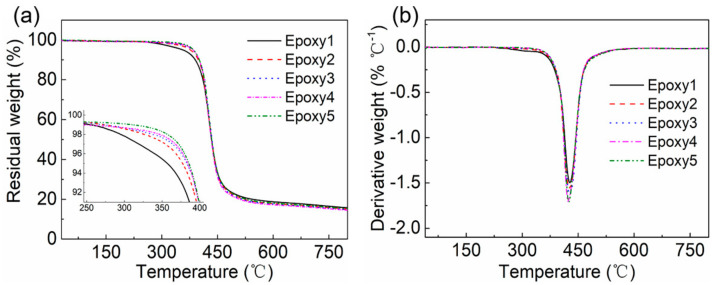
(**a**) Thermogravimetric Analysis (TGA) and (**b**) derivative thermogravimetric (DTG) curves of epoxy resins from room temperature to 800 °C under a nitrogen atmosphere with a heating rate of 10 °C/min.

In order to determine the appropriate dissolution and recycling temperature which was far lower than the pyrolysis temperature of epoxy resins, the thermal stability of epoxy-anhydride networks was determined using TGA (Figure 6 and Table 3). The epoxy resins with higher crosslink density showed higher thermal stability [71,72]. Specifically, the *T*_5%_ (the temperature at 5% weight loss) values ranged from 357 °C for *r* = 1:1.00 to 385 °C for *r* = 1:2.00, while the *T*_10%_ (the temperature at 10% weight loss) values ranged from 390 °C to 401 °C. The lower thermal stability of the resin with *r* = 1:1.00 could be associated with the degradation of unreacted epoxy monomer in the network. The derivative thermogravimetric (DTG) curves showed that the *T*_max_ (the temperature corresponding to the maximum weight loss rate) of each sample was ~425 °C. In the subsequent dissolution and recycling experiments, the temperature of 140–200 °C was used to avoid the influence of thermal degradation.

### 3.3. Swelling and Dissolution of Epoxy Resins

**Figure 7:**
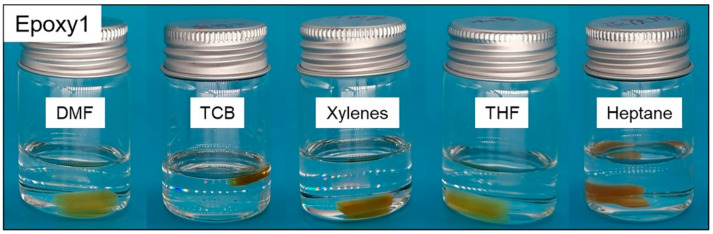
The appearance of Epoxy1 after being immersed in N,N-dimethylformamide (DMF), TCB, xylenes, tetrahydrofuran (THF), and heptane at ambient temperature for 96 h.

**Figure 8:**
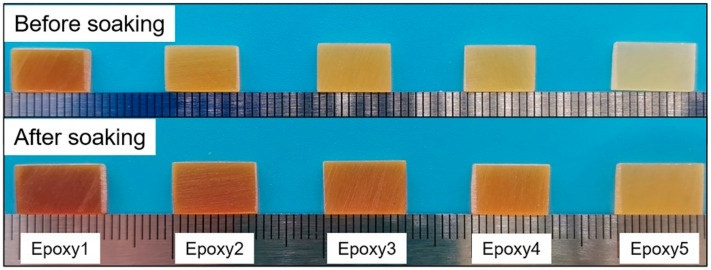
The appearance of epoxy resins with different crosslink densities before and after being soaked in TCB at 180 °C for 12 h.

In order to explore the solubility of epoxy resins, Epoxy1 was immersed in DMF, TCB, xylenes, THF, and heptane respectively at ambient temperature for 96 h. The color of these solutions did not change (Figure 7), indicating the good solvent resistance of epoxy resins at room temperature. Further, epoxy resins with different crosslink densities were soaked in TCB at 180 °C for 12 h, and the appearance of samples before and after immersion was presented in Figure 8. The samples only swelled but did not degrade, indicating that the crosslinked DGEBA/GA/DMP-30 had good solvent resistance at high temperatures. All samples were then taken out and dried in the oven at 180 °C for 12 h. The mass changes during the process were recorded in Table 4. The resins with higher crosslink densities had better solvent resistance, and the slight mass loss was caused by the dissolution of the unreacted monomers in solvents [51]. The results were consistent with that of FTIR and thermal stability.

**Figure 9:**
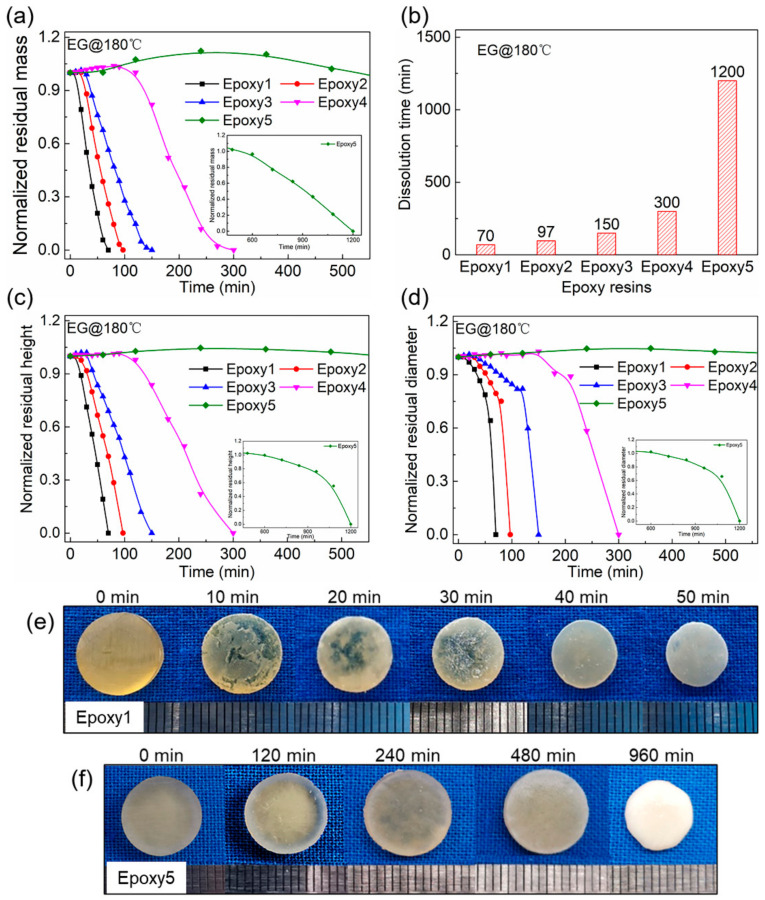
The dissolution of epoxy resins in EG at 180 °C: (**a**) Normalized residual mass of samples with different contents of GA as a function of time. The inset figure shows the normalized residual mass evolution of Epoxy5 after being immersed in EG for 480 min; (**b**) The time required for the complete dissolution of samples; (**c**) Normalized residual height of epoxy resins as a function of time; (**d**) Normalized residual diameter of epoxy resins as a function of time; (**e**) The appearance and size evolution of Epoxy1; (**f**) The appearance and size evolution of Epoxy5.

The dissolution of epoxy resins with different crosslink densities in EG at 180 °C is characterized (Figure 9). The residual mass of the sample during the dissolution process is normalized by the initial mass. It should be noted that the residual mass here is the total mass of the remaining sample containing EG molecules in the network. As shown in Figure 9a, the sample mass increased slightly at first due to the diffusion of EG into the networks. The soaking time, at which the mass loss began to occur, was delayed with the increase of crosslink density. For example, Epoxy1 began to lose weight after ~10 min of soaking, while that for Epoxy5 was ~480 min. The dissolution rate for all the samples decreased from ~5.5 × 10^−3^ g/min (Epoxy1) to ~4.5 × 10^−4^ g/min (Epoxy5) with the increase of GA dosage. Epoxy1 can be completely dissolved after soaking in EG at 180 °C for ~70 min, while that for Epoxy5 with the highest crosslink density was ~20 h (Figure 9b). The thickness and diameter changes of samples were also recorded during the dissolution, and the results were basically consistent with the variation of mass (Figure 9c,d). The height kept a relatively uniform decrease while the diameter decreased slowly first and then rapidly. Figure 9e,f showed the size and appearance evolution of Epoxy1 and Epoxy5 after being soaked in EG for different times at 180 °C, respectively. The cylindrical shape of samples remained unchanged, indicating that the dissolution of the epoxy resin was a typical surface erosion mode [32,40,41]. The swollen layer was evident during the dissolution of Epoxy5, and the swelling mass reached ~12 wt% after being immersed in EG for ~4 h. The influence of crosslink density can be explained according to the three processes that determine the dissolution rate of epoxy resins: the diffusion of EG molecules into networks, the cleavage of polymer chains due to the TERs between hydroxyl groups and ester bonds, and the diffusion of broken chain segments into EG solvent [6,32,39]. For the resins with high crosslink density, it is difficult for EG to diffuse into the network, and the resistance of short sections sliding out of the network increased. The above results indicated that even the DGEBA/GA/DMP-30 systems with high crosslink density and insufficient hydroxyl groups, the EG solution can provide abundant hydroxyl groups to break the ester bonds, leading to the complete dissolution of polymers.

**Figure 10:**
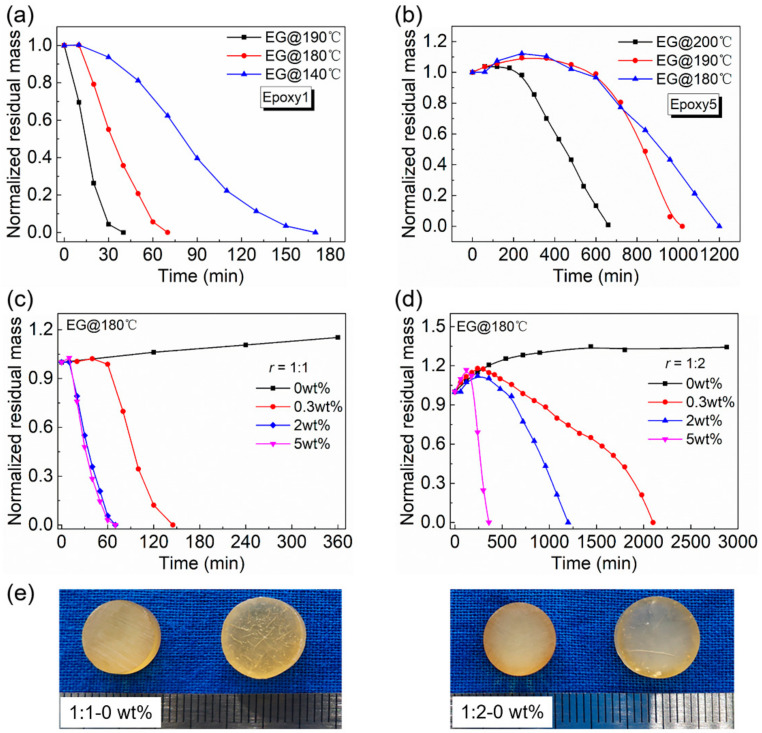
The influence of temperature and DMP-30 content on the dissolution of epoxy resins: (**a**) The normalized residual mass of Epoxy1 as a function of time after immersing in EG at different temperatures; (**b**) The normalized residual mass of Epoxy5 as a function of time after immersing in EG at different temperatures; (**c**) When *r* = 1:1, the normalized residual mass of epoxy resins with different contents of DMP-30 as a function of time; (**d**) When *r* = 1:2, the normalized residual mass of epoxy resins with different contents of DMP-30 as a function of time; (**e**) The appearance and size changes of samples without DMP-30 after being soaked in EG at 180 °C for 48 h.

Figure 10 showed the effect of the temperature and DMP-30 content on the dissolution of epoxy resins. A higher temperature led to the fast degradation of epoxy resins. For example, when the temperature increased from 140 °C to 190 °C, the dissolution rate of Epoxy1 increased from ~3.1 × 10^−3^ g/min to ~6.1 × 10^−3^ g/min (Figure 10a). When the temperature increased from 180 °C to 200 °C, the complete dissolution time required for Epoxy5 with the highest crosslink density reduced almost half (Figure 10b). The high temperature promoted the entire process of epoxy dissolution, which meant a higher diffusion rate and TER rate. To investigate the influence of catalyst content, the samples with different DMP-30 contents (0.3 wt%, 2 wt%, and 5 wt%) were prepared. In addition, the epoxy resins without DMP-30 were also synthesized as control. It was observed that increasing the amount of DMP-30 brought forward the initiation of dissolution and accelerated the depolymerization of epoxy resins (Figure 10c,d). For example, when *r* = 1:2, the dissolution rate increased significantly from ~1.8 × 10^−4^ g/min to ~2.5 × 10^−3^ g/min as the content of DMP-30 shifted from 0.3 wt% to 5 wt%. When *r* = 1:1 and DMP-30 content was 5 wt% (Figure 10c), the dissolution rate did not increase significantly because the etherification reaction between epoxy and DMP-30 could not be ignored (Figure S3) [51,53]. The ether bonds formed during the curing were not conducive to the dissolution of epoxy resins. The effect of DMP-30 content was mainly attributed to the increase of the TER rate with the catalyst content [73]. For the network without tertiary amine moiety, no dissolution was observed even after being soaked in EG for 48 h (Figure 10e). After absorbing EG, the final weight of samples with *r* = 1:1 and 1:2 reached 128 wt% and 134 wt%, respectively. In combination with the swelling results of epoxy resins in other organic solvents (Figure 7 and Figure 8), the results indicated that the TER between hydroxyl and ester bonds under the catalysis of tertiary amine was the main reason for the dissolution of epoxy resins.

### 3.4. Reuse of Decomposed Epoxy Oligomer

**Figure 11:**
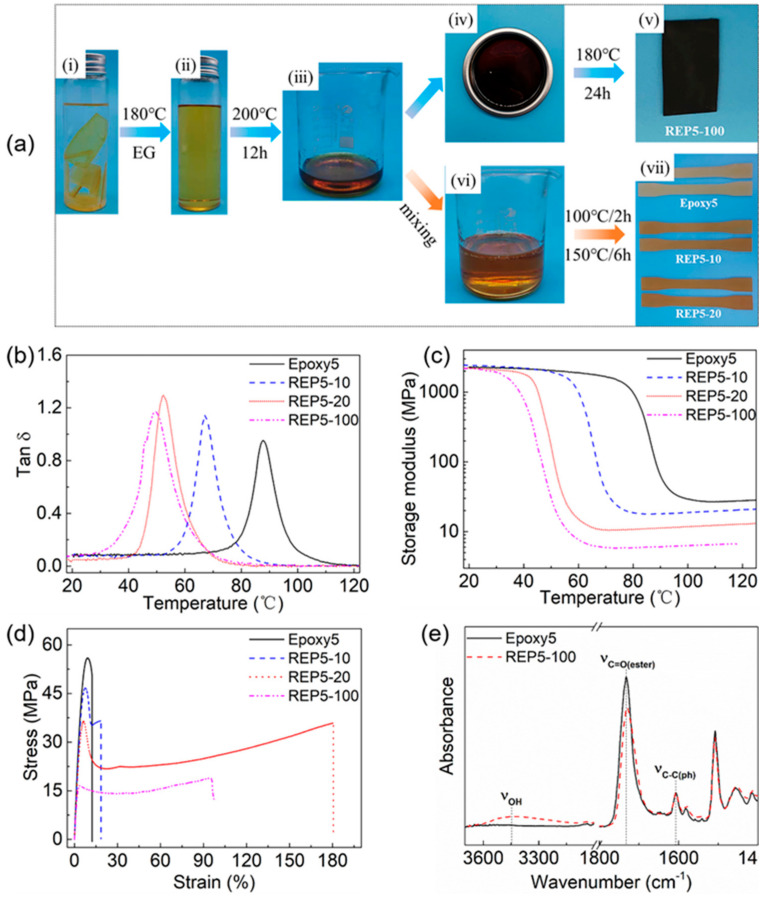
(**a**) The experimental process of the dissolution, recycling, and reuse of Epoxy5: The epoxy samples are immersed in EG (i) and heated at 180 °C for ~20 h to be completely dissolved (ii). The degradation solution is heated at 200 °C in an open environment to obtain the decomposed epoxy oligomer (DEO) (iii). The DEO is poured into the mold (iv) and heated at 180 °C for ~24 h to get a repolymerized sample (v). The DEO is mixed with DGEBA, GA, and DMP-30 (vi) to prepare new epoxy resins (vii). REP5-20 is the epoxy material containing 20 wt% DEO; (**b**) Tan δ curves of epoxy resins containing different amounts of DEO; (**c**) Storage modulus of epoxy resins with different DEO contents; (**d**) Tensile stress-strain curves of epoxy resins with different DEO contents; (**e**) FTIR spectra of fresh Epoxy5 and repolymerized sample.

The decomposed epoxy oligomer (DEO) reclaimed through evaporating excess EG can be reused to prepare new epoxy resins. Taking Epoxy5 as an example (Figure 11a), the fractured epoxy samples after the tensile tests were immersed in EG and heated at 180 °C for ~20 h to be completely dissolved. The yellow and transparent degradation solution was then heated at 200 °C to evaporate the residual EG. There were two ways to reuse the obtained DEO. On the one hand, the DEO was poured into the mold and heated at 180 °C for ~24 h to get a repolymerized sample, which was dark due to the oxidation. On the other hand, the DEO can also be used as raw materials to participate in the preparation of new epoxy resins, in which REP5-20 meant the epoxy material containing 20 wt% DEO. The new resin samples were brown due to the addition of DEO. Figure 11b,c showed the effect of DEO amount on the dynamic mechanical properties of epoxy samples. Only one tan δ peak was observed for all samples, indicating the adequate miscibility between DEO and epoxy-anhydride systems. Both *T*_g_ and rubbery modulus decreased with the increase of the DEO amount. For example, the *T*_g_ and rubbery modulus of REP5-20 were 52.26 °C and 11.94 MPa, respectively, which were much lower than those of Epoxy5. The crosslink density calculated by equation (1) showed a remarkable decrease with the increase of DEO content: 2.91 mmol/cm^3^ for Epoxy5 vs 1.28 mmol/cm^3^ for REP5-20 (Table 5). Figure 11d showed the tensile stress-strain curves of the cured resins with different DEO contents. It could be seen that compared with the original resin (Epoxy5), the samples containing DEO showed different tensile behavior, which underwent obvious yield and necking phenomenon during the tensile process. When 10 wt% DEO was added (REP5-10), after reaching the yield point, the stress decreased with the increase of strain, and the specimen necked locally then broke. The elongation at break was slightly higher than that of Epoxy5. When 20 wt% DEO was added (REP5-20), the local necking extended to the whole specimen gradually, and the stress increased slowly with the growth of strain. Finally, the sample fractured after a large deformation. The tensile test process of REP5-20 was recorded in Figure S4. All the mechanical properties of the reprocessed resins were summarized in Table 5. It could be seen that the moduli of resins containing DEO were close to that of the virgin resin, but the tensile strength decreased significantly with the increase of DEO. Specifically, the elastic modulus ranged from 1168.54 MPa for Epoxy5 to 971.01 MPa for REP5-20, while the tensile strength ranged from 55.39 MPa to 36.48 MPa. The elongation at break of samples increased significantly after adding the DEO, which even reached 195% for REP5-20. The resin prepared by 100 wt% DEO had lower *T*_g_, rubbery modulus, tensile strength, but comparable elastic modulus, which was 49.60 °C, 6.27 MPa, 18.81 MPa, and 1008.26 MPa, respectively. REP4-10 and REP4-20 exhibited similar performance (Figure S5). As shown in Figure 11e, abundant hydroxyl groups existed in the repolymerized epoxy resins, and the intensity of the ester bond peak was reduced. The residual EG molecules reduced the crosslink density of new resins, resulting in the lower *T*_g_, rubbery modulus, and tensile strength. Besides, the addition of DEO incorporated a large number of branch and dangling chains, including unreconnected ester bonds and broken irreversible bonds into networks, which made the polymer chains more flexible, resulting in higher elongation at break [41]. Common epoxy resins are usually rigid and brittle, which greatly limits their industry application [74,75]. The addition of DEO can increase the toughness of epoxy resins, which is of great significance to the modification of epoxy resins.

## 4. Conclusions

In this work, DGEBA, GA, and DMP-30 were used to synthesize recyclable high-performance epoxy resins. Compared with early degradable epoxy vitrimers, the resins prepared in this work exhibited a higher *T*_g_ (87.73 °C), tensile strength (65.59 MPa), and elastic modulus (1390.24 MPa). The preparation and recycling of these resins did not need other expensive and toxic organic salt or strong base catalysts such as Zn(Ac)_2_, TBD, etc. DMP-30 played a dual role of participating in the curing process and activating the TERs during the recycling. Five kinds of epoxy resins with different crosslink densities were prepared by adjusting the ratio of epoxy monomer and anhydride. The thermal and mechanical properties of the resins varied with GA content. The influences of crosslink density, DMP-30 content, and temperature on the dissolution rate were then studied. Specifically, epoxy resins with higher crosslink density had a lower dissolution rate in EG. The increase of DMP-30 content and temperature could advance the initiating point of dissolution and speed up the decomposition of epoxy resins. Finally, the decomposed epoxy oligomer reclaimed through evaporating excess EG was used as a reactive ingredient to prepare new epoxy resins with high modulus and ductility. The method proposed in this work is low-cost and easy to implement, which provides a new strategy for the modification, classification, and recovery of epoxy resins. Further work is needed to explore whether the recycling mechanism is suitable for other epoxy-anhydride-tertiary amine catalyst systems. This work may impact the applications of epoxy resins in detachable adhesives, removable electronic encapsulation, non-destructive surface coatings, recyclable composite materials, and so on.

## Figures and Tables

**Table 1:** Formulations of diglycidyl ether of bisphenol A (DGEBA)/-glutaric anhydride (GA)/ 2,4,6-tris(dimethylaminomethyl)phenol (DMP-30) curing systems.

Name	*r*^1^ (mol:mol)	DMP-30 ^2^ (wt%)
Epoxy1	1:1.00	2
Epoxy2	1:1.25	2
Epoxy3	1:1.50	2
Epoxy4	1:1.75	2
Epoxy5	1:2.00	2

^1^ The stoichiometric ratio of epoxy group in DGEBA and acyl group in GA; ^2^ The weight percentage of DMP-30 in the curing system.

**Table 2:** Effect of the stoichiometric ratio of the epoxy group and acyl group (*r*) on properties of epoxy resins.

System	*r*	*T*_g_ (°C)	Rubbery Modulus (MPa)	Crosslink Density (mmol/cm^3^)	Elastic Modulus (MPa)	Tensile Strength (MPa)	Elongation at Break (%)
Epoxy1	1:1.00	46.39	5.28	0.57	1155.05 ± 9.87	42.24 ± 1.89	5.16 ± 0.33
Epoxy2	1:1.25	54.70	10.03	1.06	1373.79 ± 34.23	52.11 ± 1.80	5.62 ± 0.23
Epoxy3	1:1.50	65.06	15.23	1.57	1390.24 ± 82.83	65.59 ± 2.11	9.22 ± 0.31
Epoxy4	1:1.75	74.60	21.04	2.12	1342.37 ± 41.81	62.33 ± 0.63	9.93 ± 0.83
Epoxy5	1:2.00	87.73	29.86	2.91	1168.54 ± 35.09	55.39 ± 0.52	11.15 ± 0.84

**Table 3:** Effect of the stoichiometric ratio of epoxy group and acyl group on the thermal stability of epoxy resins.

System	*r*	*T*_5%_^1^ (°C)	*T*_10%_^2^ (°C)	*T*_max_^3^ (°C)
Epoxy1	1:1.00	357	390	427
Epoxy2	1:1.25	377	398	427
Epoxy3	1:1.50	383	401	426
Epoxy4	1:1.75	384	401	423
Epoxy5	1:2.00	386	401	425

^1^ The temperature at 5% weight loss; ^2^ The temperature at 10% weight loss; ^3^ The temperature at the maximum weight loss rate.

**Table 4:** The mass changes of epoxy resins after being immersed in organic solvents and dried.

System	*r*	Organic Solution	Conditions	*m*_s_/*m*_0_^1^ (%)	*m*_d_/*m*_0_^2^ (%)
Epoxy1	1:1.00	DMF	25 °C for 96 h	181.3	95.9
		TCB		103.1	99.9
		xylenes		101.8	99.8
		THF		170.5	95.3
		heptane		100.7	100.0
Epoxy1	1:1.00	TCB	180 °C for 12 h	159.8	95.5
Epoxy2	1:1.25			155.9	95.9
Epoxy3	1:1.50			146.3	98.8
Epoxy4	1:1.75			139.3	99.7
Epoxy5	1:2.00			134.8	99.6

^1^ The ratio of swollen mass (*m*_s_) to original mass (*m***_0_**); ^2^ The ratio of dried mass (*m*_d_) to original mass.

**Table 5:** Effect of the DEO content on properties of reprocessed epoxy resins.

System	*T*_g_ (°C)	Rubbery Modulus (MPa)	Crosslink Density (mmol/cm^3^)	Elastic Modulus (MPa)	Tensile Strength (MPa)	Elongation at Break (%)
Epoxy5	87.73	31.13	2.91	1168.54 ± 35.09	55.39 ± 0.52	11.15 ± 0.84
REP5-10 ^1^	67.10	20.48	2.10	1018.39 ± 25.73	46.88 ± 0.19	16.78 ± 1.67
REP5-20 ^2^	52.26	11.94	1.28	971.01 ± 2.59	36.48 ± 0.03	195.36 ± 14.97
REP5-100 ^3^	49.60	6.27	0.67	1008.26 ± 18.08	18.81 ± 2.12	101.79 ± 5.71

^1^ The epoxy resin containing 10 wt% DEO; ^2^ The epoxy resin containing 20 wt% DEO; ^3^ The epoxy resin containing 100 wt% DEO.

## Data Availability

The data presented in this study are available on request from the corresponding author.

## Supplementary Materials

The following are available online at https://www.mdpi.com/2073-4360/13/2/296/s1, Table S1: The dissolution time of Epoxy1 in different alcohol solvents; Table S2: Summary of the reported degradable epoxy resins based on transesterification reactions between ester bonds and hydroxyl groups; Figure S1: FTIR spectra of Epoxy5 cured at 150 °C for different hours; Figure S2: Stress relaxation curves of Epoxy1 at different temperatures; Figure S3: The etherification reaction between DGEBA and DMP-30; Figure S4: The uniaxial tensile test process of REP5-20; Figure S5: Properties of reprocessed Epoxy4 containing different amounts of DEO.
