# Supplementary material for: Recyclable High-Performance Epoxy-Anhydride Resins with DMP-30 as the Catalyst of Transesterification Reactions
Source: Polymers (Basel). 2021 Jan 18;13(2):296. doi: 10.3390/polym13020296 (PMC7831910; doi:10.3390/polym13020296)
Supplement: Supplementary file 1 [file polymers-13-00296-s001.pdf]

polymers
MDPI

Supporting Information

Recyclable High-Performance Epoxy-Anhydride Resins with DMP-30 as the Catalyst of Transesterification Reactions

Wenzhe Zhao [1], Le An [1,*] and Shujuan Wang [2]

[1] State Key Laboratory for Strength and Vibration of Mechanical Structures, School of Aerospace Engineering, Xi'an Jiaotong University, Xi'an 710049, China; wenzhe0301@stu.xjtu.edu.cn
[2] School of Chemistry, Xi'an Jiaotong University, Xi'an 710049, China; shujuanwang@mail.xjtu.edu.cn
* Correspondence: anle2018@mail.xjtu.edu.cn

Citation: Zhao, W.; An, L.; Wang, S. Recyclable High-performance Epoxy-Anhydride Resins with DMP-30 as the Catalyst of Transesterification Reactions. *Polymers* **2021**, *13*, 269. https://doi.org/10.3390/polym13020296

Received: 8 December 2020
Accepted: 15 January 2021
Published: 18 January 2021

Publisher's Note: MDPI stays neutral with regard to jurisdictional claims in published maps and institutional affiliations.

Copyright: © 2021 by the authors. Licensee MDPI, Basel, Switzerland. This article is an open access article distributed under the terms and conditions of the Creative Commons Attribution (CC BY) license (http://creativecommons.org/licenses/by/4.0/).

Content

Table S1. The dissolution time of Epoxy1 in different alcohol solvents.

Table S2. Summary of the reported degradable epoxy resins based on transesterification reactions between ester bonds and hydroxyl groups.

Figure S1. FTIR spectra of Epoxy5 cured at 150 °C for different hours.

Figure S2. Stress relaxation curves of Epoxy1 at different temperatures.

Figure S3. The etherification reaction between DGEBA and DMP-30.

Figure S4. The uniaxial tensile test process of REP5-20.

Figure S5. Properties of reprocessed Epoxy4 containing different amounts of DEO.

Table S1. The dissolution time of Epoxy1 in different alcohol solvents.

Solvent	Boiling point(°C)	Chemical structure	Dissolution temperature (°C)	Dissolution time
Ethanol	78	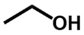	140	2 days
Ethylene glycol	197	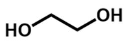	140<br>180	170 min<br>70 min
2-Ethyl-1-Hexanol	183-186	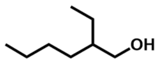	180	4 days
Propylene Glycol	187	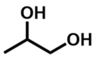	180	90 min

Table S2. Summary of the reported degradable epoxy resins based on transesterification reactions between ester bonds and hydroxyl groups.

Starting materials	T_g (°C)	Rubbery modulus (MPa)	Elastic modulus (MPa)	Tensile strength (MPa)	$T_{5\%}$ (°C)	Stress relaxation time (s)
DGEBA/fatty acids/$Zn(Ac)_2$ [1]	30	2.5	3.8	3.0	350	600 at 200 °C
DGEBA/GA/$Zn(Ac)_2$ [2]	63	15	728.3	37.7	390	109 at 220 °C
BPA/TGDDM/DA [3]	20	2.9	3.3	3.2	Not reported	3200 at 200 °C
Eu-EP/SA/$Zn(Ac)_2$ [4]	53	Not reported	Not reported	25	310	128 at 200 °C

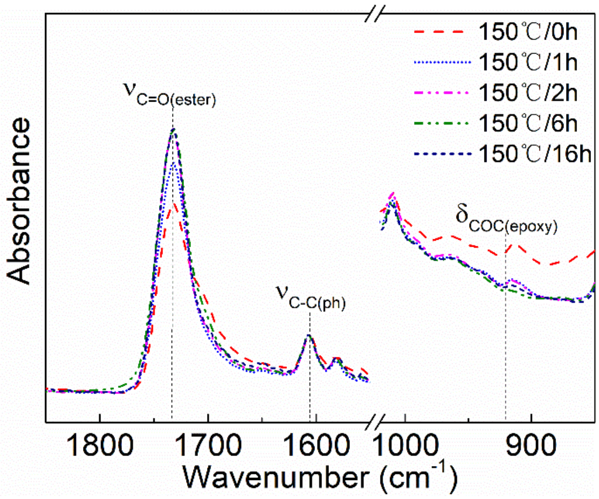

Figure S1. FTIR spectra of Epoxy5 cured at 150 °C for different hours.

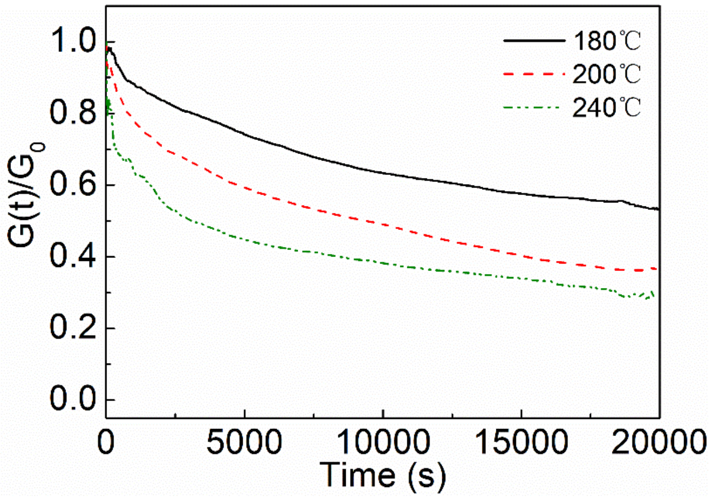

Figure S2. Stress relaxation curves of Epoxy1 at different temperatures. Stress relaxation tests of epoxy resins were performed on an MCR 302 rheometer (Anton Paar, Austria) equipped with a 25 mm circular parallel plate. The circular specimen with a thickness of ~1 mm and a diameter of 25mm was heated to the designed temperature and equilibrated for 10 min. A 1.5 N normal force and a 1% strain were then applied to the sample, and the deformation was maintained during the measurement. The relaxation modulus of epoxy resin as a function of time was recorded.

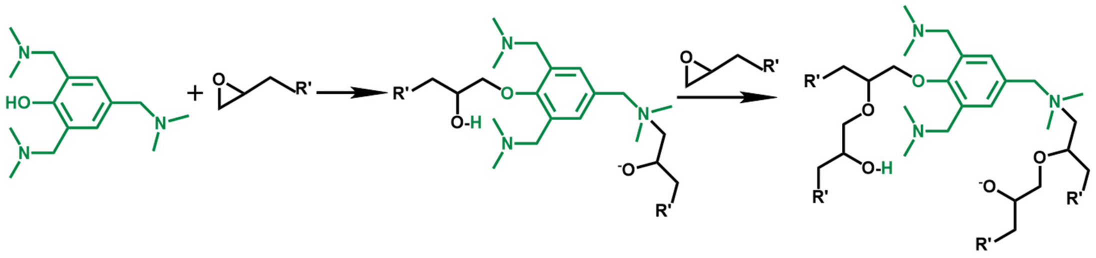

Figure S3. The etherification reaction between DGEBA and DMP-30.

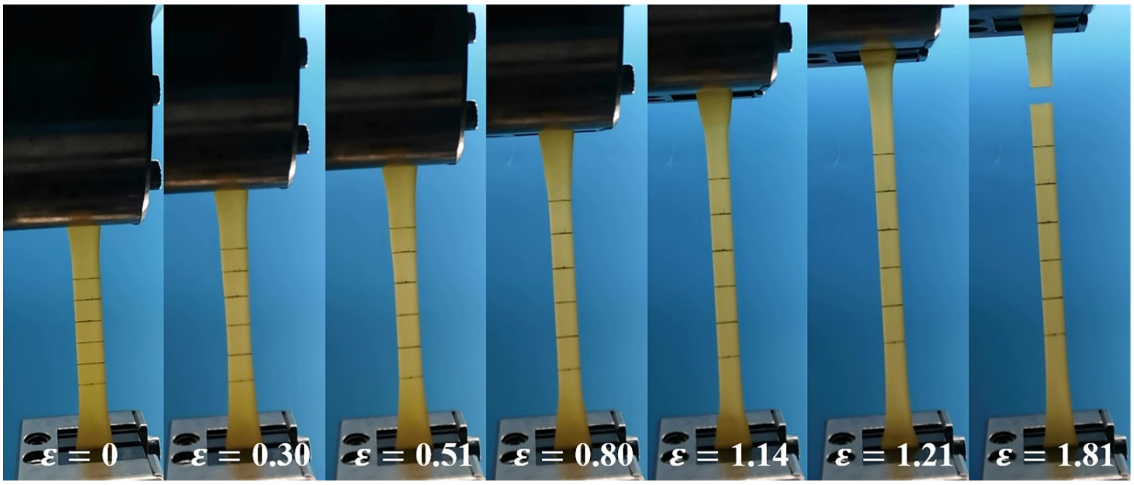

Figure S4. The uniaxial tensile test process of REP5-20.

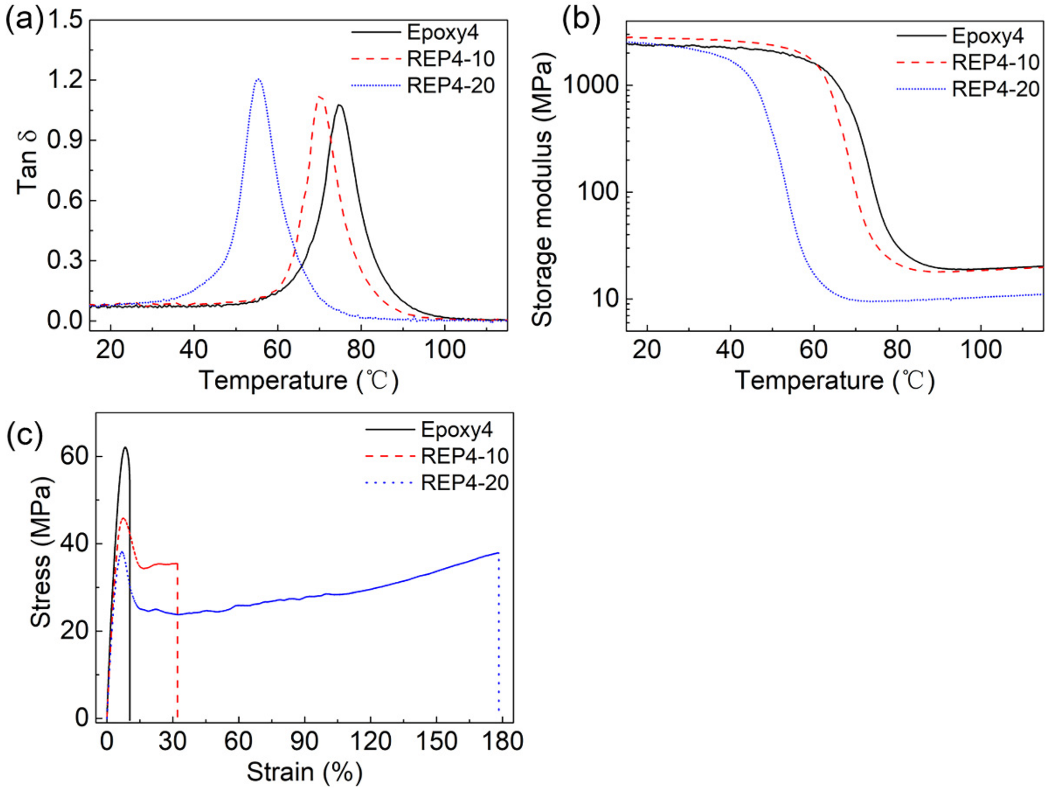

Figure S5. Properties of reprocessed Epoxy4 containing different amounts of DEO: (**a**) Tan δ curves as a function of temperature. (**b**) Storage modulus curves as a function of temperature. (**c**)Tensile stress-strain curves.

References

1. Yu, K.; Taynton, P.; Zhang, W.; Dunn, M.L.; Qi, H.J. Influence of stoichiometry on the glass transition and bond exchange reactions in epoxy thermoset polymers. *RSC Adv.* **2014**, *4*, 48682-48690, doi:10.1039/c4ra06543c.
2. Wang, D.; Li, L.Y.; Ke, H.J.; Xu, K.L.; Lu, S.; Gong, W.H.; Zhang, H.; Wang, G.Y.; Zhao, Y.M.; Zhao, N. Preparation and Properties of Recyclable High-performance Epoxy Resins and Composites. *Acta Polym Sin* **2020**, *51*, 303-310, doi:10.11777/j.issn1000-3304.2019.19167.
3. Li, Y.; Liu, T.; Zhang, S.; Shao, L.; Fei, M.; Yu, H.; Zhang, J. Catalyst-free vitrimer elastomers based on a dimer acid: robust mechanical performance, adaptability and hydrothermal recyclability. *Green Chemistry* **2020**, *22*, 870-881, doi:10.1039/c9gc04080c.
4. Liu, T.; Hao, C.; Wang, L.; Li, Y.; Liu, W.; Xin, J.; Zhang, J. Eugenol-Derived Biobased Epoxy: Shape Memory, Repairing, and Recyclability. *Macromolecules* **2017**, *50*, 8588-8597, doi:10.1021/acs.macromol.7b01889.
